# Measuring the Mental Health-Care System Responsiveness: Results of an Outpatient Survey in Tehran

**DOI:** 10.3389/fpubh.2015.00285

**Published:** 2016-01-27

**Authors:** Setareh Forouzan, Mojgan Padyab, Hassan Rafiey, Mehdi Ghazinour, Masoumeh Dejman, Miguel San Sebastian

**Affiliations:** ^1^Department of Public Health and Clinical Medicine, Umea University, Umea, Sweden; ^2^Social Determinants of Health Research Centre, University of Social Welfare and Rehabilitation Sciences, Tehran, Iran; ^3^Ageing and Living Conditions Programme, Centre for Population Studies, Umea University, Umea, Sweden; ^4^Department of Social Work, Umea University, Umea, Sweden; ^5^Social Welfare Management Research Center, University of Social Welfare and Rehabilitation Sciences, Tehran, Iran; ^6^Department of Mental Health, Bloomberg School of Public Health, Johns Hopkins University, Baltimore, MD, USA

**Keywords:** responsiveness, mental health care, outpatient, social status, Iran

## Abstract

As explained by the World Health Organization (WHO) in 2000, the concept of health system responsiveness is one of the core goals of health systems. Since 2000, further efforts have been made to measure health system responsiveness and the factors affecting responsiveness, yet few studies have applied responsiveness concepts to the evaluation of mental health systems. The present study aims to measure responsiveness and its related domains in the mental health-care system of Tehran. Utilizing the same method used by the WHO for its responsiveness survey, responsiveness for outpatient mental health care was evaluated using a validated Farsi questionnaire. A sample of 500 public mental health service users in Tehran participated and subsequently completed the questionnaire. On average, 47% of participants reported experiencing poor responsiveness. Among responsiveness domains, confidentiality and dignity were the best performing factors while autonomy, access to care, and quality of basic amenities were the worst performing. Respondents who reported their social status as low were more likely to experience poor responsiveness overall. Attention and access to care were responsiveness dimensions that performed poorly but were considered to be highly important by study participants. In summary, the study suggests that measuring responsiveness could provide guidance for further development of mental health-care systems to become more patient orientated and provide patients with more respect.

## Introduction

The Universal Declaration of Human Rights and its related conventions and guidelines, which have been ratified by governments globally, contain a wide range of health-related rights. These include the right to health and health care, and particularly the right of individuals with physical and mental disabilities to the highest quality health services (1). When governments proceed to promote and protect public health, their actions must adhere to certain criteria to ensure that people are treated in ways that respect their rights and respond to their legitimate expectations (2). With the intention to assess the extent to which the health-care systems perform close to user expectations (based on their experiences with the health system), in 2000, the concept of responsiveness was developed and operationalized by the World Health Organization (WHO) (3). Responsiveness is defined as a measure of how individuals are treated and the environment in which they are treated and includes eight domains (4). Certain aspects of human rights, such as respecting patients’ autonomy, dignity, confidentiality, and choice of health care as well as client orientation aspects, such as the quality of basic amenities, prompt attention, and access to social support are covered in the concept of responsiveness.

Responsiveness becomes particularly relevant when considering the mental health-care system (5). Compared to other health service users, mentally ill patients are at the greatest risk for having their rights violated because of the characteristics of the mental disorders and the stigma attached to them (6). At the same time, research shows the importance of an active interaction between the mental health system and the service users to achieve better mental health-care outcomes and reduce delays in service referral (7, 8).

Beginning in the late 1980s, in order to achieve the highest attainable level of mental health care, the Islamic Republic of Iran began integrating mental health services into primary health care. At the village or neighborhood level in urban areas, community health workers are in charge of mental health-care responsibilities, including active case finding and referral. At primary care centers, trained general practitioners provide mental health care as part of their general health-care responsibilities. In case of complex mental health problems, general practitioners refer patients to district or provincial health centers, which are supported by university mental health hospitals (9). The strong existing ties between medical education and health sectors facilitate the integration process around the country.

The way in which mental health services were organized after their integration into public health care positively affected the coverage of treatment for people with diverse mental disorders (10). However, despite the increase in coverage shown by national and regional surveys (11, 12), information about the quality of mental health care is lacking.

The application of the WHO responsiveness concept could contribute to assessing some of the quality aspects of the mental health-care system in Iran. This study aims to assess how the domains of responsiveness are performing in the mental health-care system of Tehran. In addition, two interrelated objectives will be explored: (i) Are the perceptions of responsiveness different by sociodemographic characteristics? and (ii) Which responsiveness domains are most important to service users? Is it those with good or poor performance?

## Materials and Methods

### Setting

In some ways, the health system structure and organization in the Islamic Republic of Iran is unique. At the national level, the Ministry of Health and Medical Education (MoHME) is responsible for health service delivery through planning, designing, and implementing health policies as well as monitoring and supervising health-related activities for the public and private sectors (8). Yet, the MoHME implements health policies and plans via medical universities across the country. There is at least one medical university in every province. The president of a medical university is the highest health authority in the province and is assigned by and reports to the MoHME. The medical universities are in charge of public health activities, health-care provision in public facilities, and medical education. Health-care and public health services are provided through a nation-wide network including a referral system that starts at the household/health post level in the periphery, goes through secondary-level health centers in districts and at provincial and capital level, ends at university hospitals.

As in many large cities, the provision of mental health care in Tehran is complex. A combination of public, private, and special services such as military hospitals and clinics as well as services related to semi-private schools of medicine (e.g., Azad medical university) are involved in mental health-care provision. Most people, especially those from poor neighborhoods, depend on public mental health services because of the high costs of private care.

### Study Population

The survey was conducted in Tehran, the capital of Iran, between January and April 2013. In Tehran, mental health services are organized in terms of catchment areas. Each of the four public medical universities provides inpatient and outpatient mental health services for a defined catchment area. These services are provided through mental hospitals and nine affiliated outpatient clinics. However, service users can freely choose to be referred to any of the mental health services.

A non-random sample of 500 mentally ill patients referring to 27 mental health service providers in nine public outpatient clinics distributed in different city regions (north, south, east, west, and central) was selected. Private psychiatric clinics were excluded. The number of participants assigned to each clinic was proportional to the total number of patients attending the clinics during the previous 3 months.

Service users diagnosed as mentally ill based on a psychiatric evaluation record (the psychiatrists were blinded to the study). The study participants were recruited after being initially approached by the interviewers with regard to their willingness to participate. Inclusion criteria were (1) being an adult (18–65 years old), (2) receiving outpatient care during the past 12 months, and (3) being mentally capable of following the interview according to their clinical record. Type of mental disorder was not considered an inclusion criterion since experiences that mental health patients have with services relate more to the services functioning than to the patient’s current diagnosis (13, 14). Service users older than 65 years were not included in our study because previous studies showed that at least about 10% of them have different level of age-associated memory impairment (15).

Trained external interviewers with a bachelor degree in psychology conducted face-to-face interviews. Interviewers explained the procedure of the interview to the participants and obtained their written consent. Participants were asked about the outpatient mental health-care services that they had experienced during the last 12 months; interviews lasted between 45 and 50 min and were strictly anonymous. To ensure subjects were not interviewed twice, each record was labeled with a code. The Ethical Committee and Research Council of the University of Social Welfare and Rehabilitation Sciences in Iran approved the study. In addition, administrative permission was obtained from the Medical Universities in the study area. All study participants were evaluated by a psychiatrist prior to the interview. Based on the psychiatrist-written clinical reports, participants were in the remission phase of their mental disorder and capable of making a decision. The nature and purpose of the study was explained to each participant; then, individual informed consent was confirmed by participants with a signature or a left thumbprint. Before the interview, participants were informed that the completion of the questionnaire was voluntary and their identification would be protected, as the data files were anonymous. Participants were informed of their right to withdraw from the study at any time. Participants gave permission to audiotape the interview session.

### The Instrument

The responsiveness concept developed by the WHO was applied in this study (16). In a previous qualitative study, we evaluated the applicability of this concept to mental health care in Iran. With some modifications, the concept was proved to suit Iranian mental health service users’ expectations (17). A new domain of effective care was added; the domain of prompt attention was divided into two new domains (attention and access to care), and the domains choice of health-care providers and autonomy were combined into one domain (Table 1) (18).

**Table 1:** **Domains covered in the WHO and Farsi responsiveness questionnaires**.

Domains in WHO questionnaire	Domains in Farsi questionnaire	Definition
Prompt attention (convenient travel, short waiting times)	Attention	Close and affable dialog between mental health workers and patients, attend to and respond to the patients with deep understanding, having enough time to ask questions about mental health problem or treatment, proactive and careful follow-up of the process of treatment by service providers; mental health-care providers show they understand how patients feel about their problem
	Access to care	Acceptable care provided as soon as needed by patient
Dignity (respectful treatment, communication)	Dignity	Showing respect when treating patients, not being stigmatized when dealing with service providers, patient problems, and complaints are taken seriously, to recognize patients’ individual needs and characteristics
Clear communication (listening, enough time for questions, clear explanations)	Clear communication	To provide patients with understandable information about their problem and to provide information about patient problems in a comprehensible manner
Autonomy (involvement in decisions)	Autonomy	Services and providers can be chosen freely, to be able to participate in therapeutic decisions and processes, equal patient/provider relationship
Choice of health-care provider		
–	Effective care	To provide practical advice in congruence with patient norms and values, continuity of care across services and sectors, to provide care by the same familiar person, to provide services commensurate with costs such as time and money
Confidentiality (to handle patients’ information confidentially)	Confidentiality	To handle patients’ information confidentially
Quality of basic amenities (surroundings)	Quality of basic amenities	To be treated in clean, informal, and friendly places

In accordance with the WHO health system responsiveness questionnaire (19) and the findings of our previous qualitative study (17), a Farsi version of the mental health system responsiveness questionnaire was adapted to suit the mental health-care system in Iran (18). Classic psychometric criteria of the Farsi version of the questionnaire have been measured and its feasibility, reliability, and validity tested previously (18). The questionnaire consisted of 40 questions representing eight domains. The domain “access to social support” was excluded from the questionnaire since inpatient cases were not included in this study. In addition, to measure the importance of the domains, participants were asked to identify the domain they felt was the most and the least important in mental health care.

### Data Analysis

In accordance with WHO’s approach in the Multicountry Survey Study on Health and Responsiveness (MCSS), we scored responsiveness in each domain based on the “rating” question, which was asked only after participants had answered a series of detailed “report” questions related to the relevant domain. For the rating questions, the responses were categorized as 5 (very bad), 4 (bad), 3 (moderate), 2 (good), and 1 (very good). A further summary score for “overall responsiveness” was obtained by calculating the average scores across all the eight domains. The responsiveness outcomes were then dichotomized into good responsiveness (combining the *very good* and *good* responses) and poor responsiveness (combining the *moderate*, *bad*, and *very bad* responses) (20). Multivariable logistic regression was used to assess the odds ratios of poor performance of overall responsiveness and its related domains in relation to sociodemographic characteristics of the participants. These characteristics included age, sex, education level (primary or <5 years of education, intermediate level 5–12 years, and higher education >12 years), employment, and subjective social status (SSS). SSS was recorded based on how people perceived their relative position in the social hierarchy (i.e., low, middle, and high). Because the distribution of SSS was asymmetric among study participants, the middle social position (52.7%) was chosen as the reference group. We applied chi-square test to check the bivariate association between overall poor responsiveness and sociodemographic characteristics. All analyses were performed using STATA version 12.0 and *p*-values <0.05 were considered significant (21).

## Results

### Study Group

A total of 500 participants aged 18–65 years (mean = 36.4, SD = 12 years) were enrolled in the study. Among them, 38% were females and 62% males. Approximately 24% had 5 years or less of formal education and 28.7% were unemployed. All participants revealed that they used the services more than once during the past 6 months, and 96% had used services more than twice in the last 6 months. The majority of participants (52.7%) indicated that they belonged to the middle social status, and 92.8% of participants had access to medical insurance. Details of the sociodemographic characteristics of the study participants are presented in Table 2.

**Table 2:** **Sociodemographic characteristics of the study group and *poor* overall responsiveness rating**.

Age group (years)	Participants (%)	*Poor* overall responsiveness rating (%)	*p-*Value
<25	17.7	53.7	
25–35	33.4	50.9	
36–45	26.2	44.7	
46–55	14.2	46.7	
56 and more	8.5	31.7	
			0.16
Gender			
Female	38	43.5	
Males	62	49.0	
			0.26
Subjective social status			
Low	43.1	55.0	
Middle	52.7	39.5	
High	4.2	47.6	
			0.004
Education			
Primary level	24.1	40.7	
Intermediate level	60.6	50.0	
Higher education level	15.3	42.7	
			0.17
Working status			
Employed	55.7	44.6	
Unemployed	28.7	52.2	
Retired + disabled	15.6	42.7	
			0.27

### Responsiveness Performance

On average, 47% of participants reported experiencing *poor* responsiveness. Table 2 shows the distribution of participants reporting *poor* responsiveness among the different sociodemographic groups. Youngest participants (53.7%) reported worst responsiveness than did older participants (31.7%). In addition, more participants with low SSS scored responsiveness as poor compared to those with high and middle SSS. Approximately half of the participants with intermediate educations or who were unemployed reported poor responsiveness for services.

**Figure 1:**
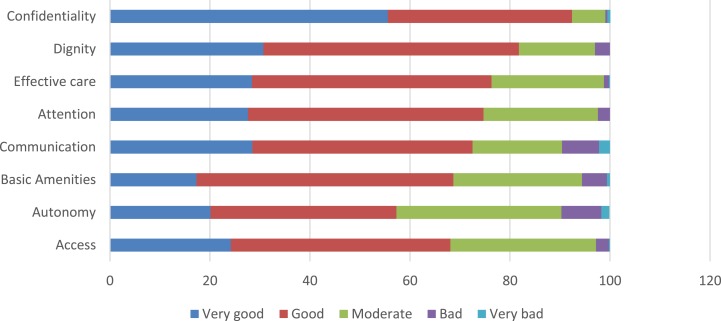
**Percentage of participants rating responsiveness domains**.

Figure 1 shows the proportion of participants who reported responsiveness as *very bad*, *bad*, *moderate*, *good*, and *very good* in all domains. The best-performing domains (*very good* and *good*) were confidentiality (92.4%) and dignity (81.8%). The worst performing (*moderate*, *bad*, and *very bad*) domains were autonomy (42.7%), access to care (31.9%), and quality of basic amenities (31.3%).

In order to examine the relationship between the poor responsiveness rating and the sociodemographic characteristics of participants, a multivariable logistic regression analysis was performed. Overall, as well per domain, SSS was the only characteristic that showed differences in rating responsiveness (Table 3). Respondents who reported themselves as belonging to a lower social status were more likely to experience overall poor responsiveness (OR = 2.2; 95% CI = 1.5–3.3). Regarding the different responsiveness domains, participants who reported themselves as belonging to a lower social status were about three times more likely to experience poor access to care (OR = 3.2; 95% CI = 2.1–4.9), poor effective care (OR = 2.9; 95% CI = 1.8–4.8), and poor dignity (OR = 2.8; 95% CI = 1.6–4.8). Lower social status was also statistically associated with experiences of poor communication (OR = 2.5; 95% CI = 1.6–3.9), poor attention (OR = 2.2; 95% CI = 1.4–3.5), and poor autonomy (OR = 1.8; 95% CI = 1.2–2.8).

**Table 3:** **Percentage and odds ratios of *poor* responsiveness (overall and per domain) in respect to subjective social status (SSS)**.**^a^**

Responsiveness and its domains	% Poor			Odds ratio^a^ (95% CI)		
	Middle SSS	Low SSS	High SSS	Middle SSS	Low SSS	High SSS
Access	21.3	45.8	23.8	1	3.2 (2.1–4.9)	0.9 (0.3–2.8)
Communication	21.0	36.0	24.0	1	2.5 (1.6–3.9)	1.3 (0.4–3.9)
Confidentiality	7.2	7.6	14.3	1	1.4 (0.6–3)	1.9 (0.4–7.3)
Dignity	12.9	23.4	33.3	1	2.8 (1.6–4.8)	2.9 (1.0–8.2)
Attention	19.9	31.8	28.6	1	2.2 (1.4–3.5)	1.5 (0.5–4.2)
Autonomy	36.6	49.8	42.9	1	1.8 (1.2–2.8)	1.4 (0.5–3.5)
Effective care	16.9	32.2	19.0	1	2.9 (1.8–4.8)	1.0 (0.3–3.3)
Quality of basic amenities	28.6	33.6	42.9	1	1.5 (0.9–2.3)	1.6 (0.6–4.2)
Overall responsiveness	39.5	55.0	47.6	1	2.2 (1.5–3.3)	1.3 (0.5–3.4)

*^a^The models are adjusted for age, gender, occupation, and education*.

### Importance of Domains and Performance

**Figure 2:**
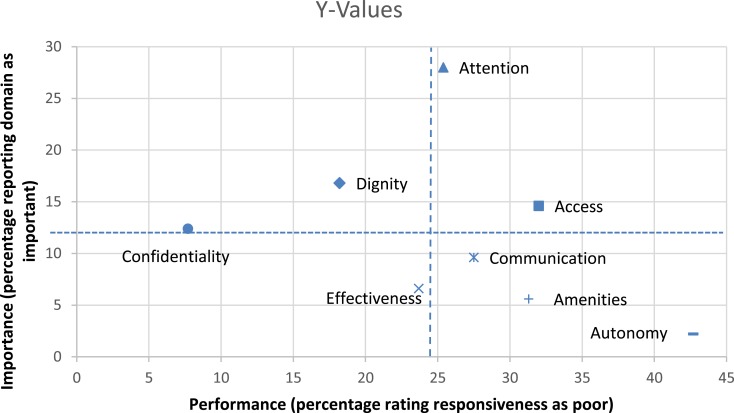
**Responsiveness by domains in relation to domain importance**.

The importance of the responsiveness domains in relation to domain performance is presented in Figure 2. The majority of respondents named attention, dignity, access, and confidentiality as the most important domains. However, the access dimension score was among the lowest in terms of performance and the score for attention performance was not good. Only the dignity and confidentiality dimensions scored high both in importance and in performance. Autonomy, quality of basic amenities and clear communication performed poorly but were considered as highly important.

## Discussion

To our knowledge, this is the first study exploring the responsiveness of the mental health-care system in Iran. Previously, only one study from Germany focused on the responsiveness of the mental health-care system (13). However, using the original form of the WHO measure, three previous studies nationally assessed general health-care responsiveness among specific groups of service users (22–24). One of those studies was conducted in Mashhad City, located in North East Iran and investigated the responsiveness of general public and private hospitals (22); the other two were performed in Tehran (23, 24). One of these studies assessed health system responsiveness in a relatively low socioeconomic district of the city (23), while the other measured responsiveness in children’s health care (24).

Our study showed that confidentiality was the best-performing domain in outpatient mental health care. This result is in line with the German study on mental health responsiveness as well as the studies from Tehran (13, 23). This finding indicates that the mental health system in Tehran has been able to build an atmosphere of trust for patients. On the other hand, this finding may be due to the fact that it is sometimes difficult for patients to be aware of the extent to which their personal information is handled confidentially. In addition, our previous qualitative study on mental health responsiveness showed that the service users have uncertainty about the limits of secrecy (17).

Dignity was the second best-performing domain in our study. This finding is also supported in previous responsiveness studies (13, 23, 25). In addition, our previous qualitative study indicated that a significant number of participants had a positive experience of being treated respectfully and not being stigmatized when referring to mental health services (17). This finding indicates that service users are treated with respect regarding their dignity when interacting with the health system in Iran, and in this case, there is no graphic difference between the health system in general and the mental health subsystem.

Autonomy, access to care, and quality of basic amenities were poor-performing domains. It is noteworthy that in our study, autonomy combines the domains of choice and autonomy used in the WHO questionnaire. In this respect, our findings are very similar to the German study on mental health responsiveness. In mental health care, there are indeed fewer opportunities for free choice and service user autonomy. This may be due to the fact that some mentally ill patients may have poor insight into their disorder and also suffer from some degree of impairments in their rational thinking. Therefore, for these patients, active participation in the process of decision making is difficult and complex. On the other hand, there is a specific desire among mental health service users to participate in mental health-care decisions, and the more they recover, the more they want to participate (26, 27). Furthermore, our qualitative study showed that even some information about the medication are provided by the doctors, but there is no consenting for medication and patients’ opinions about the medication and their side effects were not taken seriously (17). In addition, lack of sufficient health facilities as well as unfamiliarity of service providers with methods to increase participation of mental health service users, including shared decision making and transparency of mental health reports, can lead to poor autonomy.

Access to care was the second poor-performing domain. The definition of this domain in our study was very similar to the domain of prompt attention used in the WHO questionnaire. In this regard, our results are different from the German study (13) but very similar to the ones from Tehran (23). The very centralized organization of mental health facilities, as well as a lack of sufficient mental health funding and staff, might negatively influence access to care (28, 29). Improvement of the access scores appears to be resource-dependent; however, a reengineering of the patient referral process especially in big cities, such as Tehran, could undoubtedly be effective in increasing access to services.

Regarding perceptions of responsiveness among different sociodemographic groups, the German study reported that outpatient care was perceived differently depending on respondents’ income and education (13). However, in our study, the only sociodemographic variable associated with poor responsiveness was lower SSS. This could be due to the fact that SSS represents a combination of various markers of socioeconomic status and thus might reflect an individual’s social position more accurately (30). To explain possible reasons for the SSS disparities in health system responsiveness, two groups of reasons can be considered (31): (i) factors related to differential access to quality health services, particularly among poor districts of the city and (ii) service user providers-related factors. The unfair distribution of mental health facilities and human resources in Tehran, especially among suburban poor regions of the city, might partially explain the poor experience of users from low social status. Equity in access to the public health services is therefore needed. In relation to service user provider factors, a systematic review of studies exploring patients’ social position and the doctor–patient communication showed that patients from lower social status were less involved in the decision-making process and had more difficulties in understanding the medical information provided by the physician (32). At the same time, health service providers tend to perceive patients of lower social status more negatively compared with those of higher status (33). Others have also shown that regardless of patient communication behavior, physicians are more skeptical about apprehension of medical information by low-income people (34). These findings are highly relevant to our study results where lower social status participants reported more experiencing poor effective care, attention, dignity, and communication.

There is no statistically significant difference in responsiveness between males and females and between different age groups. This finding is consistent with the result of studies in health system responsiveness in Iran and the German study on mental health system responsiveness (23–25). This finding can be explained by the fact that outpatient mental health care in public facilities in Tehran is uniform.

In terms of the importance of the responsiveness domains, attention, dignity, and access to care received higher scores. This finding is also similar to a study among mentally handicapped children in Tehran (24) and a study of patient expectations in Iran that showed how the quality of interpersonal relationships is an important aspect of health service users’ expectations (35).

Performance of responsiveness domains in relation to the importance given to them in our study showed that attention and access to care do not perform well despite their importance for service users. Access to care seems to be a core expectation in general health care as well as mental health care (13, 23). The previous qualitative study of responsiveness among mental health service users also indicates that the majority of participants’ statements were related to this domain and almost all of them expected a warm and sincere approach from service providers. The complaints about poor performance of these domains may be related to shortage of human resources and facilities as well as unbalanced distribution of services (36).

## Conclusion

This is the first time that mental health system responsiveness has been measured in Iran. It represents the actual experiences of the service users when they come in contact with the mental health system. In conclusion, this study showed that dignity and confidentiality were well-performing domains, while autonomy, quality of basic amenities, and access were poor-performing domains. Improvement of all these poor-performing domains is dependent on resources. In addition, attention and access to care, which were rated high in importance and poor in performance, could be priority areas for intervention and reengineering of referral systems and admission processes. The role of the SSS in responsiveness should be further studied.

The need to assess responsiveness and its domains in the delivery of mental health services is not only important for good health practice but also for the provision of health and health-care rights (1). The findings of this study point out the strengths and weaknesses of the mental health system in Iran regarding responsiveness and establish the baseline for a future monitoring system.

### Study Limitations

Our study had a few limitations. The cross-sectional study design did not permit investigation of the cause and effect relationship between our independent variables and responsiveness. In addition, this study did not include inpatient service users. Access to social support could not be measured either. Finally, although a different dichotomization of the responsiveness outcome might have resulted in other associations, we decided to follow the guidelines provided by WHO.

## Author Contributions

SF: contributions to the conception or design of the work, analysis and interpretation of data, drafting the work, revising it critically, final approval of the version to be published, agreement to be accountable for all aspects of the work in ensuring that questions related to the accuracy or integrity of any part of the work are appropriately investigated and resolved. MP: analysis and interpretation of data, revising it critically, final approval of the version to be published, agreement to be accountable for all aspects of the work in ensuring that questions related to the accuracy or integrity of any part of the work are appropriately investigated and resolved. HR, MG, and MD: contributions to the conception or design of the work, revising it critically, final approval of the version to be published, agreement to be accountable for all aspects of the work in ensuring that questions related to the accuracy or integrity of any part of the work are appropriately investigated and resolved. MS: contributions to the conception or design of the work, interpretation of data, drafting the work, revising it critically, final approval of the version to be published, agreement to be accountable for all aspects of the work in ensuring that questions related to the accuracy or integrity of any part of the work are appropriately investigated and resolved.

## Conflict of Interest Statement

The authors declare that the research was conducted in the absence of any commercial or financial relationships that could be construed as a potential conflict of interest.
